# The Chemistry Development Kit (CDK) v2.0: atom typing, depiction, molecular formulas, and substructure searching

**DOI:** 10.1186/s13321-017-0220-4

**Published:** 2017-06-06

**Authors:** Egon L. Willighagen, John W. Mayfield, Jonathan Alvarsson, Arvid Berg, Lars Carlsson, Nina Jeliazkova, Stefan Kuhn, Tomáš Pluskal, Miquel Rojas-Chertó, Ola Spjuth, Gilleain Torrance, Chris T. Evelo, Rajarshi Guha, Christoph Steinbeck

**Affiliations:** 1 Department of Bioinformatics - BiGCaT, NUTRIM, Maastricht University, 6200 MD Maastricht, The Netherlands; 2 NextMove Software Ltd, Cambridge, CB4 0EY UK; 3 Department of Pharmaceutical Biosciences, Uppsala University, 751 24 Uppsala, Sweden; 4 AstraZeneca, Innovative Medicines & Early Development, Quantitative Biology, Möndal, Sweden; 5 Ideaconsult Ltd, A. Kanchev 4, 1000 Sofia, Bulgaria; 6 Department of Informatics, University of Leicester, Leicester, UK; 7 Whitehead Institute for Biomedical Research, 455 Main Street, Cambridge, MA 02142 USA; 8 Química Clínica Aplicada, 43870 Amposta, Spain; 9 4 Hanway Place, W1T 1HD London, UK; 10 National Center for Advancing Translational Sciences, 9800 Medical Center Drive, Rockville, MD 20850 USA; 11 Institute for Inorganic and Analytical Chemistry, Friedrich-Schiller-University, Lessingstr. 8, 07743 Jena, Germany

**Keywords:** Java, Cheminformatics, Bioinformatics, Metabolomics, Depiction

## Abstract

**Background:**

The Chemistry Development Kit (CDK) is a widely used open source cheminformatics toolkit, providing data structures to represent chemical concepts along with methods to manipulate such structures and perform computations on them. The library implements a wide variety of cheminformatics algorithms ranging from chemical structure canonicalization to molecular descriptor calculations and pharmacophore perception. It is used in drug discovery, metabolomics, and toxicology. Over the last 10 years, the code base has grown significantly, however, resulting in many complex interdependencies among components and poor performance of many algorithms.

**Results:**

We report improvements to the CDK v2.0 since the v1.2 release series, specifically addressing the increased functional complexity and poor performance. We first summarize the addition of new functionality, such atom typing and molecular formula handling, and improvement to existing functionality that has led to significantly better performance for substructure searching, molecular fingerprints, and rendering of molecules. Second, we outline how the CDK has evolved with respect to quality control and the approaches we have adopted to ensure stability, including a code review mechanism.

**Conclusions:**

This paper highlights our continued efforts to provide a community driven, open source cheminformatics library, and shows that such collaborative projects can thrive over extended periods of time, resulting in a high-quality and performant library. By taking advantage of community support and contributions, we show that an open source cheminformatics project can act as a peer reviewed publishing platform for scientific computing software.

**Graphical abstract:**
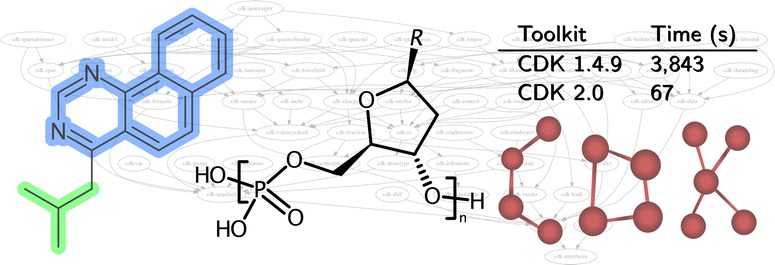
CDK 2.0 provides new features and improved performance

**Electronic supplementary material:**

The online version of this article (doi:10.1186/s13321-017-0220-4) contains supplementary material, which is available to authorized users.

## Background

The open source cheminformatics community has made significant steps forward recently [1] as evidenced by the growing number of tools and underlying toolkits, along with the usage of these software components in a variety of applications. The Chemistry Development Kit (CDK) is one of the tools developed under the aegis of the Blue Obelisk, a movement promoting Open Data, Open Source, and Open Standards in chemistry [1, 2]. The CDK providing data structures to represent chemical concepts along with methods to manipulate such structures and perform computations on them. Previously documented CDK versions have been widely adopted [3, 4]. Use of the CDK ranges from inclusion of CDK functionality in wrapper platforms such as Cinfony [5], incorporation within the R environment (rcdk [6]), and as plugins for Taverna [7], KNIME [8], Cytoscape (ChemViz2 [9]), and for Microsoft Excel (LICSS [10]). In contrast to scenarios that have made CDK functionality available in larger systems, a number of projects have employed the CDK as a general cheminformatics toolkit. Examples include jCompoundMapper [11], ScaffoldHunter [12, 13], OMG [14], PaDEL [15], ChemDes [16], ReactPRED [17], SMSD [18–20], WhichCyp [21], MetaPrint2D [22], MetFrag [23], and the IUPHAR/BPS Guide to Pharmacology [24], BRENDA [25] and QSAR DataBank [26] databases. A number of such tools were initially developed using older versions of the CDK and are updated to new releases as they are made available. Examples include Bioclipse [27, 28] and AMBIT [29–31]. The CDK has also played a role in a number of chemical studies, such as finding the maximally bridging rings in chemical structures [32], prediction of organic reactions [33], and bioactivities of compounds [34].

While the CDK has purported to be a general purpose cheminformatics toolkit, older versions were designed by a community with specific applications in mind, primary among them being structure elucidation. In addition, an implicit goal of previous versions was to have the CDK serve as an educational resource to enable students of cheminformatics to understand the underlying algorithms. This resulted in certain functionalities, such as molecular fingerprinting [35, 36], receiving more attention than others, such as stereochemistry. The outcome was significant variance in performance and features throughout the toolkit.

The growth of open source software over the last 10 years is evidence of the ability of communities of developers to develop systems and processes that lead to high quality software systems for long term use. The CDK is no different. The adoption of automatic build systems and quality control methodologies such as unit testing, automated source code validation, and peer review by fellow developers have greatly improved the stability of the library. While it has slowed development somewhat, it has allowed for cleaning up interdependencies between modules of functionality, and importantly, has improved the scalability of the development model. This has resulted in significant new functionality in core application programming interfaces (APIs) while maintaining the quality of code depending on those core APIs.

Examples of new features supported by the improved development model include InChI functionality [37], greatly improved ring detection algorithms [38], improvements to the core atom type perception module that now covers a much more comprehensive set of elements, charge states and radical species than previous versions, a more comprehensive fingerprinting API, new depiction functionality, and many speed and stability improvements.

## Implementation and results

This section describes the specifics of new APIs and improvements to pre-existing methods that are available in the latest CDK. We then discuss how we have improved and formalized the development model for the project using unit testing, code review and guidelines for handling version control. Finally we report on the availability of binary distributions of the library, allowing users to include specific modules (and their dependencies) of the CDK in their own projects (as opposed to developers who work on the CDK library itself).

### New APIs and improved implementations

We here outline various new and improved APIs in the CDK library since the two previous publications in 2003 and 2006 [3, 4].

#### Atom typing

Atom type perception is core cheminformatics functionality: the atom types describe chemical features of atoms, such as the number of neighbors, possible formal charges, (approximate) hybridization, electron distribution over orbitals and so on. However, previous versions of the CDK implemented atom type perception as part of different algorithms, resulting in duplicated and sometimes divergent typing schemes. As a result it was cumbersome to add new atom types and implement support for new charged and radical species in a consistent manner.

**Fig. 1:**
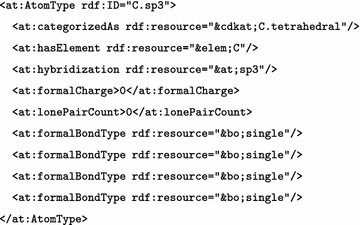
Atom type information specified for a $sp^3$-hybridized carbon

This CDK version has a new, centralized atom typing framework, removing the perception of atom types from various algorithms. This allows for a consistent and extensive typing scheme, that can be also be tested independently of other code. The new code defines the atom types using a list that specifies for each type the element symbol, hybridization, formal charge, number of lone pairs, and an enumeration of the bond orders (see Fig. 1). This list of properties captures the information needed for the various algorithms in the CDK. For example, hybridization information can be used in certain aromaticity models (see later), and the lone pair information is needed for resonance structure calculation needed, for example, for Gasteiger $\pi$-charges.

A reference implementation, CDKAtomTypeMatcher, has been written in such a way that perceives these atom types, and validates the perception automatically against the properties defined by the ontology. This class handles a variety of types of missing information, as commonly resulting from various (file) formats; for example, it can handle undefined hydrogen counts and undefined double bond positions if hybridization information is provided instead. That makes the perception code flexible but also more complex. Alternative algorithms for atom typing have not been explored. This reference implementation can be used on a single atom:

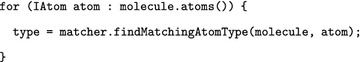



And on a full molecule, in which case the list of types is ordered in the same order as the atoms in the molecule object:

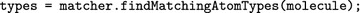



#### Stereochemistry

Previous versions of the API represented stereochemistry in different ways. This hindered interconversion between and within file formats. CDK v2.0 standardizes upon a new core representation and procedures have been updated or added to enable duplicate checking, pattern matching, and interconversion.

**Fig. 2:**
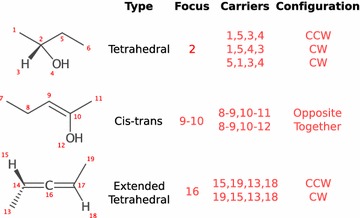
Relative storage of stereochemistry, the type and focus of stereochemistry are fixed for a given stereocenter description but the carriers and configuration are relative. The multiple rows for each stereochemistry type are different internal representation that would be considered equivalent. In the tetrahedral types, hydrogens may be suppressed in a molecular graph so the focus is reused in the carriers list as a placeholder

The preferred representation of stereochemistry is now for it to be stored at the molecule level as a StereoElement. In abstract terms a stereo element describes local geometry using a type, focus, carriers, and configuration (Fig. 2). Currently the most common types of stereochemistry are supported: Tetrahedral, Cis-trans isomerism around a double bond, and Extended Tetrahedral. Rarer types of stereochemistry, such as: Square Planar, Trigonal Bipyramidal, Octahedral, could easily be incorporated into the chosen description given sufficient demand from the community.

Along with the new stereochemistry representation, algorithms were required in several areas. Generally, a user does not need to invoke these procedures explicitly as they are called as needed within existing APIs:

- perception from 2D coordinates,
- perception from 3D coordinates,
- wedge assignment,
- graph (sub)isomorphism matching,
- SMARTS matching, and
- canonicalization.

**Fig. 3:**
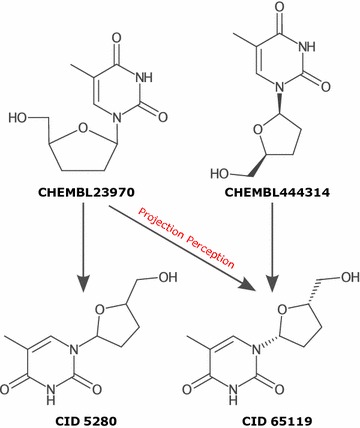
The raw input files of CHEMBL23970 and CHEMBL444314 are displayed (ChEMBL 21). Without perceiving the stereochemistry indicated by Haworth projection in CHEMBL23970, the database entries are incorrectly considered distinct. Down stream aggregation databases mirror this separation (PubChem CID 5280, CID 65119)

The perception from coordinates and wedge assignment algorithms are fundamental for conversion between formats that store stereochemistry implicitly based on coordinates (e.g. molfile,^1^ CML) and explicitly (e.g. SMILES, CML, InChI). Perception from 2D coordinates can optionally identify perspective projections, specifically: Fischer, Haworth, and Chair projections. With the perception of perspective projections enabled, database entries currently considered distinct can be merged (Fig. 3).

Pattern matching of stereochemistry with the described representation is straight forward. Given the atom–atom mapping from a query structure to a target molecule, the focus and carriers of the query stereochemistry are mapped to the target. Using the permutation parity of this mapping the configurations were compared. SMARTS matching requires some special handling for complex cases [39]. For canonicalization, a partial canonical ordering is used to assign an absolute label which can then be integrated into the ordering. The algorithms used for stereochemistry are thoroughly detailed in Chapter 6 of [40]. The perception from projections is based on an algorithm briefly described by [41].

#### Atomic and molecular signatures

An implementation has been provided of the Signature structure descriptor for molecules [42]. These act as a linear notation—like the SMILES format—for the whole molecule as well as for connected substructures rooted at a single atom. The descriptor can also be canonicalized to provide isomorphism-independent representations [43]. Signatures of depth two can be calculated for atoms with:

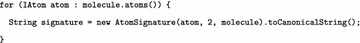



But they can also can be calculated for full molecules:

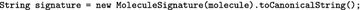



Finally, a signature fingerprint can be calculated for molecules, to allow similarity calculations. This can then be used in QSAR modeling [34, 44–49].

#### Rendering API

A new rendering API has been introduced to make the rendering code independent from Java widget toolkits. The previous code was tightly linked to the Swing toolkit, but other tools use different widget toolkits. For example, Bioclipse is based on Eclipse which uses the Standard Widget Toolkit (SWT) [27].

A second new design goal was introduced to balance between size restrictions of some use cases, such as Java applets, and the rendering functionality. In particular, some functionality, even after modularization, needed considerable parts of the CDK library, making creation of a small-sized applet unfeasible. Therefore, the rendering API was modularized to allow splitting up rendering functionality into modules, with varying CDK dependencies.

Rendering is split up into several generation steps: previous versions split up bond from atom rendering. Heteroatom symbols were simply drawn over lines representing bonds using a white rectangle to mask. A new StandardGenerator has been introduced that does bond and atom rendering at the same time. It incorporates many ideas described by Alex Clark [50, 51]. The depictions generated are of much higher quality and suitable for publication.

Moreover, a simplified high-level API has been introduced that addresses most of the common rendering needs, with the DepictionGenerator class. To depict a molecule loaded into a variable ‘benzene’ the following code can be used:

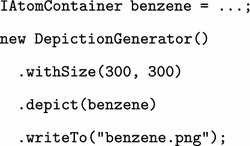



**Fig. 4:**
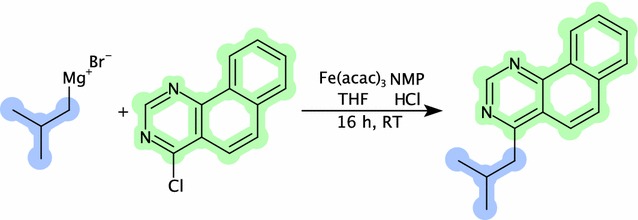
Integrated example showing the rendering and SMILES parsing functionality. Example from U.S. Patent US 2014 231770 A1 para 287

Many of the rendering options are available as parameters in the core API and as methods on the DepictionGenerator class. This includes substructure coloring, exemplified with an example reaction shown in Fig. 4. When missing, 2D coordinates are generated on the fly with the new structure diagram layout functionality.

#### Structure diagram layout

**Fig. 5:**
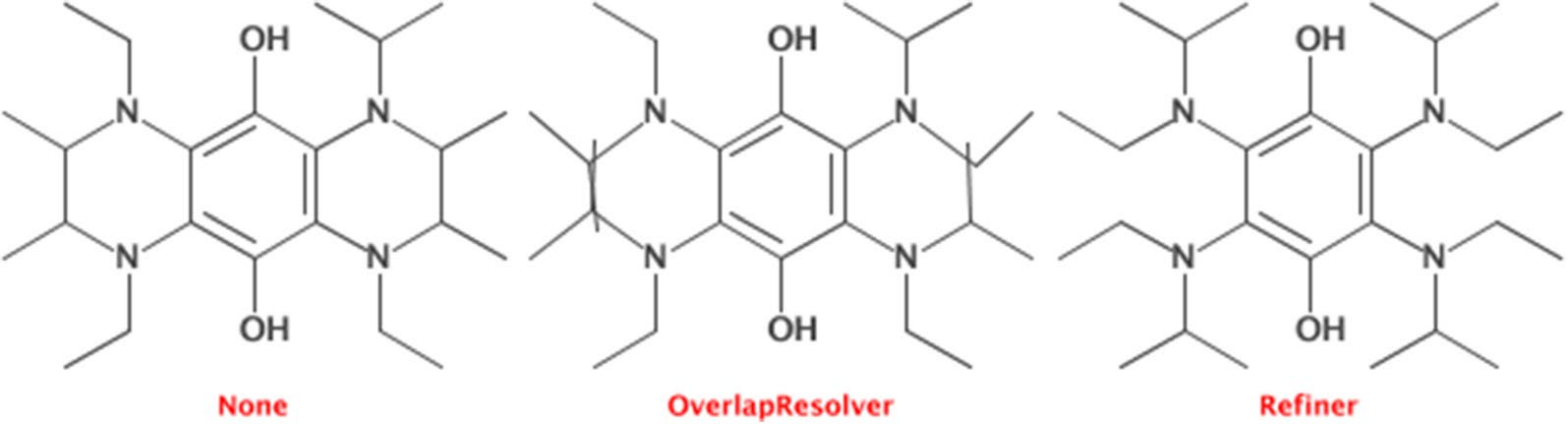
The improved structure diagram generation has improved code to solve overlap. The original SDG code used general heuristics (*left*) and the OverlapResolver would fine tune the layout to ensure atoms would not be placed at the same location (*middle*). The new SDG algorithm is able to make more rigorous changes, making the final output must more pleasing (*right*)

The structure diagram layout has been improved and the new code solves a number of long standing issues. In particular, collision avoidance has been greatly improved. Figure 5 shows a difference in output between the old code base, with and without overlap resolving, and with the new refinement based implementation [52]. Generation of 2D coordinates is done as shown below:

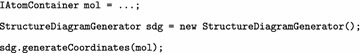



**Fig. 6:**
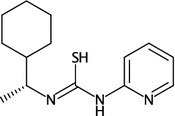
Structure diagram generation for structures with double bond and tetrahedral stereochemistry

While the API itself has not been significantly changed, the internals have been revamped. In addition to improved overlap resolution noted above, the engine appropriately handles large ring systems, maintains input stereochemistry, and makes use of a large template library. Templates are useful for laying out substructure. While previous CDK versions partially supported double bond stereochemistry the new engine is more efficient in using this information when generating 2D layouts. Furthermore, the engine assigns wedge bond information based on tetrahedral stereochemistry. These features are exemplified by the following code and the resulting layout depiction in Fig. 6:

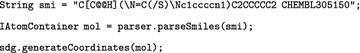



#### Molecular formula

A chemical formula is the simplest chemical representation of a compound. It defines the number of isotopes or elements that compose a compound without describing how atoms are bonded. With the rise of metabolomics it has become increasingly relevant to have full support for these in cheminformatics libraries [23, 53–56].

The CDK interfaces can handle several concepts related to chemical formulas: the formula itself, sets of formulas, chemical formula ranges, adducts, isotope containers and patterns, and rules to filter formula sets. These new tools can be used for a number of tasks, including calculating the isotopic pattern from a given chemical formula, determining the possible elemental compositions for a given mass (mass decomposition), and calculating the exact mass from a given chemical formula.

The CDK contains two algorithms for the decomposition of mass ranges into possible elemental formulas. For most inputs, a Round Robin algorithm, originally developed for the SIRIUS metabolite identification tool [57], is used. The algorithm discretizes the real-value mass decomposition problem into an integer-value knapsack problem [58]. It first computes a dynamic programming table and then backtracks within it to generate matching formulas [59, 60]. Data for the Round Robin algorithm is stored in an extended residue table [61], resulting in a low memory footprint of several kilobytes. For certain problem instances, such as very large mass values (above 400,000 Da) or mass range span larger than 1 Da, the Round Robin algorithm is not suitable and CDK falls back to an optimized full enumeration search method, originally developed as part of the MZmine 2 framework for mass spectrometry data processing [54, 55].

The following code calculates all possible chemical formulas for a given accurate mass, within allowed counts for each element:

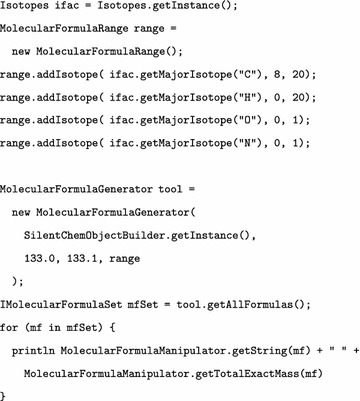



This gives the following output:

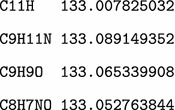



To evaluate the performance of the CDK molecular formula generator, we compared its runtimes to those of the classic, full enumeration-based HR2 formula generator [62] and those of a recently developed Parallel Formula Generator (PFG) [63] (Table 1). As inputs, we used two sets of 10,000 small (<500 Da) and 20 large (>1500 and <3500 Da) molecular mass values downloaded from the Global Natural Products Social Molecular Networking database [64]. The mass tolerance was set to 0.001 or 0.01 Da. The CDK v2.0’s Round-Robin formula generator outperformed the other methods in all cases, despite running in a single thread (PFG utilizes multiple threads). The performance gain of the Round Robin algorithm was particularly apparent when narrow mass ranges were queried (e.g. ±0.001 Da), thus showing its suitability for applications in high-resolution mass spectrometry.

**Table 1:** Evaluation of molecular formula generators

Input	Mass tolerance (±Da)	# of generated formulas			Runtime (s)		
		HR2	PFG	CDK	HR2	PFG	CDK
10,000 small masses	0.001	616,846	616,846	616,843	669	168	41
10,000 small masses	0.01	6,163,303	6,163,302	6,163,326	689	501	212
20 large masses	0.001	4,912,939	4,912,939	4,912,904	26,370	1292	177
20 large masses	0.01	49,128,811	49,128,810	49,128,815	26,587	3406	1580

The resulting formula counts and runtimes of the HR2, PFG, and CDK chemical formula generators on two different inputs with two different mass tolerance settings. For the set of small masses, 10,000 mass values in the range of 0–500 Da were randomly selected from the Global Natural Products Social Molecular Networking database [64]. For the set of large masses, 20 mass values in the range of 1500–3500 Da were randomly selected from the same database. Formulas were generated using chemical elements C, H, N, O, P, S without bounds (the allowed atom count was set to 0–10,000 for each element). All heuristic filtering rules were disabled for the purpose of the evaluation. The slight differences in the number of generated formulas were caused by different isotope masses embedded in each software and/or by rounding errors during calculation. The runtimes are average values from three independent runs performed on three different 16-core Intel Xeon 2.9 GHz CPU workstations equipped with 189 GB RAM, running Ubuntu Linux version 12.04.5 LTS and OpenJDK Java runtime version 1.7.0_101

#### SMILES parser and generator

The SMILES [65] parsing has been replaced by code from the external Beam project [66]. This BSD-licensed SMILES parser is a complete implementation of the SMILES and OpenSMILES (http://opensmiles.org/) specifications by one of the authors (including stereochemistry), and is independent of the CDK library. The SmilesParser API uses this library underneath, and the Beam API is hidden by this class. Basic usage is as follows:

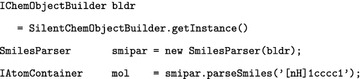



The most significant functional change here is that the SMILES parser automatically locates the positions of double bonds in de-localised aromatic systems (Kekulisation). If this invariant cannot be met the SMILES is rejected as invalid. It is possible to override this check but this is strongly discouraged as rejected molecules do not have a fixed formula or tautomer [40].

The SMILES generation API has also been simplified and made more flexible able to produce several different flavours. The SmiFlavor flags are used to control the type of SMILES generated. Historically the terms: generic, isomeric, unique, absolute have been used in other toolkits and are also supported.

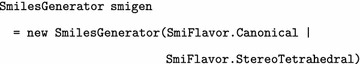



Support for ChemAxon Extended SMILES (CXSMILES) [67] layers has been added to CDK v2.0. CXSMILES provides a powerful means of including auxiliary information in a SMILES string such as 2D/3D coordinates, atom values, generic labels, repeat units, and positional variation. CXSMILES is achieving by placing additional information between pipe characters (‘|’) in the SMILES title field. Information is annotated based on the order of the atoms in the SMILES string. An example CXSMILES for a generic structure is shown below.

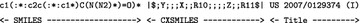



#### Substructure and SMARTS matching

Substructure matching is fundamental cheminformatics operation and plays a key role in many other functions such as fingerprint and descriptor generation, and atom typing. Since CDK v1.2, functionality has been added to handle the SMARTS query language. The SMARTS language is supported well including features such as stereochemistry, component grouping, and atom maps (to match reaction transformations). A new *Pattern* API has been added to CDK v2.0, which simplifies finding, filtering, and transforming search results. The API is immutable allowing a pattern to be initialized once and then matched against several molecules or reactions across multiple threads. During initialization the pattern is inspected so as to determine what invariants will be needed (e.g. ring size) and only required invariants are calculated. The internal matching algorithms provide a lazy iterator, such that the next match is only computed when it is needed. The API handles reactions in addition to molecules, and both can be specified as either queries or targets.

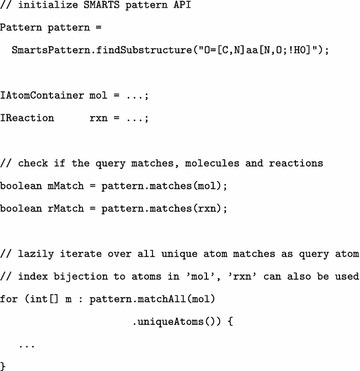



CDK v2.0 includes large improvements to algorithm efficiency. This is emphasised in the systematic benchmark of MACCS-like 166 key generation (Table 5). The efficiency improvements are a combination of optimising data structures and key molecule processing algorithms (e.g. kekulisation and aromaticity) needed before a SMARTS match can be run [40, 68, 69].

#### Ring finding

Ring finding is another key functionality in a cheminformatics library, and the CDK knows a long history of ring finding [38, 70]. Specifically, non-redundant ring sets have seen particular interest, such as the smallest set of smallest rings, for which the CDK implements two classical algorithms [70, 71]. Recent work has implemented a new, faster algorithm, allowing searching for various types of (non-redundant) ring sets [38]. These are available via the new Cycles API:

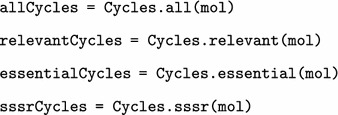



#### Aromaticity

Aromaticity has seen many definitions in the past and for cheminformatics it frequently is algorithmically defined. The outcome of an aromaticity calculation depends on a number of atom type features and heuristics, which are often ambiguously defined in the published literature. Based on the information used, several different algorithmic definitions of aromaticity can be defined. Older CDK versions had various aromaticity models implemented but the code was scattered throughout the library, resulting in an inconsistent API to compute aromaticity and a significant maintenance burden. The API was unified in the current version, resulting in three models, of which two are based on the CDK atom typer. The difference between these two models is how contributions from exocyclic double bonds are handled.

The current CDK version further generalizes the idea that aromaticity is a model, and provides an API that allows the user to select one of several aromaticity models, leading to greater interoperability with other toolkits. The new Aromaticity class allows to build a custom model by selecting and combining options. For example, to reproduce the functionality of the previous CDKAromaticity class:

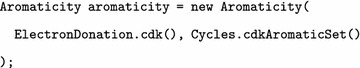



Here, the CDK model for counting donated electrons is used, along with the rings systems that were identified by the older algorithm in previous versions that was limited in the number of fused rings systems that were considered. However, an alternative aromaticity calculator that considers all possible ring systems can now be easily created with:

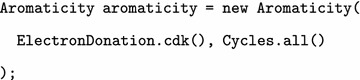



For SMARTS matching and SMILES generation a model based on Daylight [72] can be used and offers significant speed improvements to the one based on CDK Atom Types. This model has recently been documented as part of the OpenSMILES specification (http://opensmiles.org/):

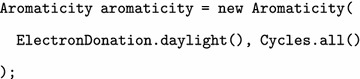



The aromaticity algorithm is straight forward, the potential electron donation is calculated for each atom as $-1$ (not aromatic), 0, 1, 2. The set of cycles provided in the constructor is then generated and each is checked for Hückel’s rule ($4n+2$).

#### CTfile format improvements

The molfile format is still very popular and despite it being a proprietary format, it has become a de facto standard. The format forms the core of the larger CTfile family which was originally developed by MDL Information Systems [73]. The current format specification is published by BIOVIA and available on request [74].

The CTAB block (connection table) of a molfile comes in two versions, V2000 and V3000. The V3000 provides several enhancements including but not limited to: removing atom and bond count limits, enhanced stereochemistry, and link nodes. For backwards compatibility V2000 is often preferred resulting in limited usage of V3000.

CDK v2.0 adds support for V3000 and has optimized and extended support for V2000. Currently these are considered separate formats requiring a user to know what version is being read beforehand. Future APIs will aim to simplify this and provide a unified reader. An overview of currently supported CTfile formats is given in Table 2.

**Table 2:** CTfile format support

Format	V2000	V3000
MOLfile	Read and write	Read and write
RXNfile	Read and write	Read
SDfile MOLfile	Read and write	Read
RGfile	Read and write	
RDfile		

CTfile Sgroups capture and organise high level information about sets of atoms and bonds [75]. There are four types of Sgroup: Display Short-cuts, Polymers, Mixtures, and Data. The most familiar Sgroups from an end user perspective are structure repeat units (e.g. bracketing) and abbreviations (Fig. 8). CDK  v2.0 adds supports for representation, reading, writing, and depiction of Sgroups.

#### New object builders

Originally, the CDK was developed as a shared library between JChemPaint [76] and Jmol [77, 78]. JChemPaint used a MVC approach with an event-passing mechanism to update the view when the model was changed. This can cause a cascade of change events being passed around. This was not always a desirable feature, especially for non-UI code. To address this, interfaces were introduced allowing multiple implementations of the core interfaces. With much code of the CDK library no longer based on the original data model, a builder is needed to create objects of that data model, such as an implementation of the IAtom. The new IChemObjectBuilders allow implementations to be created, allowing implementations of the interfaces to be instantiated without the need of explicitly referencing those implementations. This way, any algorithm implementation in the CDK can use any of the data model interface implementations.

The CDK v1.0 and v1.2 implementations of the IChemObjectBuilder had, however, one method for each data object constructor, resulting in a very large interface. Moreover, this interface API had to be updated each time a new class was introduced, and when existing methods changed and constructors were updated. To simplify the API, the new IChemObjectBuilder collapses all methods into a single method, which takes as a first parameter the class of the interface that is to be constructed. All further parameters are passed as parameters to the class constructor.

For example, to construct a new atom from its element symbol, one would write previously:

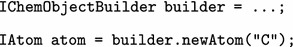



With the new builder, the code looks like:

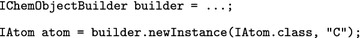



The CDK library is now mostly refactored and no longer depends on a specific implementation of the IChemObjectBuilder, allowing the user of the CDK to select a builder suitable to their software. Therefore, if software depends on event passing, then the DefaultChemObjectBuilder can be used, in most cases this isn’t needed and the SilentChemObjectBuilder is preferred resulting in a typical speed up of 10–20%:

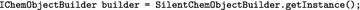



The third builder is the DataDebugChemObjectBuilder which generates debug information for all changes to the content of the data classes. This can be useful for debugging and other forms of code inspection.

#### Molecular fingerprints

Molecular fingerprints have also seen significant development in this CDK version. Previously, fingerprints were represented using the BitSet class from the Java library. While using this class allowed the use of pre-existing methods to manipulate bit strings, it keeps a vector of bits in memory. The solution was excellent for hashed, relatively small fingerprints, e.g., 1024 bits, i.e. with a $2^{10}$ indexing space (128 B). However, implementing a fingerprint designed to avoid collisions with a $2^{32}$ bit indexing space using this approach would be memory-inefficient (512 MiB). To allow for multiple fingerprint representations, a bit fingerprint interface was introduced: IBitFingerprint.

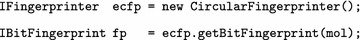



Also, although fingerprints traditionally are bit vectors a count fingerprint was also introduced making fingerprints based on integer vectors supported in CDK as well. The counts in the fingerprint then represent how often this substructure is found in the molecule it represents.

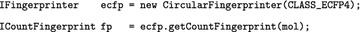



The fingerprints currently provided by the CDK are listed in Table 3.

**Table 3:** The molecular fingerprints in CDK

	Bit version	Count version	CDK version	Default Size
CircularFingerprinter [35, 86]	$\checkmark$	$\checkmark$	v2.0	1024/$2^{32}$*
EStateFingerprinter [87]	$\checkmark$		v1.2.0	79
ExtendedFingerprinter	$\checkmark$		v1.0	$1024$
Fingerprinter	$\checkmark$		v1.0	$1024$
GraphOnlyFingerprinter	$\checkmark$		v1.0	$1024$
HybridizationFingerprinter	$\checkmark$		v1.4.0	$1024$
KlekotaRothFingerprinter [88]	$\checkmark$		v1.4.6	4860
LingoFingerprinter [89]	$\checkmark$		v2.0	NA$^{\dagger }$
MACCSFingerprinter	$\checkmark$		v1.2.0	166
PubchemFingerprinter [90]	$\checkmark$		v1.4.0	881
ShortestPathFingerprinter	$\checkmark$		v2.0	1024
SignatureFingerprinter [44]	$\checkmark$	$\checkmark$	v2.0	$2^{32}$
SubstructureFingerprinter	$\checkmark$		v1.0	307

Listed are the currently available molecular fingerprint in CDK with information about whether they come as a bit and/or count version, what CDK version they were introduced in, their default size, and relevant references, where applicable

* For the CircularFingerprinter the bit version is folded to 1024 whereas the count version is unfolded

$^\dagger$ The LingoFingerprinter does not have a default size

### Improved coding standards

As the CDK library grew over the years, so did the complexity of the maintenance. The main branch frequently failed to compile and bug fixes became more onerous due to unexpected side effects. Often fixing a bug in one part of the code, broke some other code which made the incorrect assumptions about the fixed code. With the increased size of the CDK developer community, such issues were inevitable in the absence of any formal coding and testing standards.

To address these issues, we have adopted a number of coding standards. While not a comprehensive implementation of software engineering best practices, they attempt to find a balance between increasing code maintainability and being flexible enough to allow efficient code development. We appreciate the subjective nature of this statement, and some adopted guidelines have been heavily discussed and debated in the CDK community.

Arguably, perhaps the biggest factor in improved code quality is a peer review process where any functionality changing patch is required to be reviewed by one independent, senior CDK developer for the development branch, and by two reviewers for stable branches. This patch development system is supported by a number of automated validations steps as outlined below. The next sections describe some approaches the project have adopted that allows us to maintain the CDK library as it is today.

#### Stability and version identifier

Prior to CDK v2.0, the parity of the version identifier’s second digit indicated stability. Even numbers (v1.2.x, v1.4.x) indicating API stability and odd numbers (v1.3.x, v1.5.x) indicating potential API instability. Versions v1.4.x and v1.5.x were developed in parallel, where possible patches were applied to both. As the APIs diverged the amount of effort to port patches from the development but more robust v1.5.x to v1.4.x became unmanageable for the core development team. This even-odd version scheme was adopted from old Linux kernel versioning that was subsequently abandoned in 2004 for time-based releases [79].

At the time of writing the development branch is more than 3000 commits ahead of v1.4.x. As the the v1.5.x API has become stable it became time to release v1.6.x. Due to significant API changes in 2011^2^ it was felt a larger digit increment was needed. This provided the opportunity to change to a more manageable and intuitive version identifier.

From CDK v2.0 a new sequence based version scheme will be used. The version identifier indicates change significance as follows:

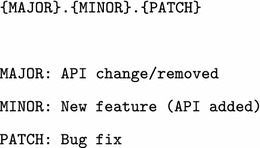



Due to limited developer resources we envision that releases will primarily increment the minor version with the occasional patch release. As per Maven convention, development versions are suffixed with -SNAPSHOT. There are no API changes from v1.5.x and v2.0.

#### Modularization

**Fig. 7:**
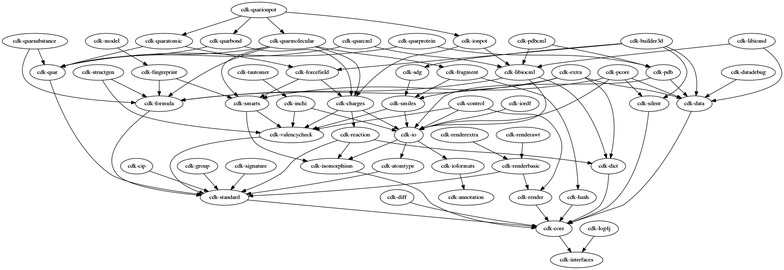
Dependencies between CDK modules. Visualization of the dependencies between CDK modules. For example, the cdk-core depends on the cdk-interfaces module. A few higher level modules have been left out: cdk-builder3dtools, cdk-legacy, and cdk-depict

One of the central approaches we have adopted, is to make the CDK more modular. The CDK assigns every class to a module, and defines dependencies between modules. For example, core modules are not allowed to depend on modules with data classes implementing the CDK interfaces; instead, they may only depend on the interfaces themselves. This ensures that dependencies are minimized. Furthermore, it also allows cherrypicking CDK functionality, reducing the number of third-party library dependencies that are needed. An overview of key modules with description, important changes, and dependencies on third-party libraries is given in Table 4 and the dependencies between the CDK modules are depicted in Fig. 7.

**Table 4:** A selection of key CDK modules with major changes

Module	Description	Major changes	Dependencies
interfaces	Interfaces for the data models		Vecmath 1.5.2
core	Core functionality		Google Guava 17.0
standard	Common functionality		
render	Graphical rendering	Redesigned to make it more modular and support Multiple widget toolkits, like AWT and SWT	
isomorphism	Isomorphism and substructure searching		
atomtype	Various non-core atom type schemes	Unified approach where atom typing is separated from other algorithms	
ioformats	Definitions of (chemical) input/output formats		
io	Readers and writers for input/output formats	The molfile reader has been rewritten and supports atom types defined in the specification	XPP3 1.1.4c
iordf	Stores data models as in the Resource Description Framework serialization formats	New	Jena 2.7.4
inchi	IUPAC International Chemical Identifier support		JNI-InChI 0.8 [37]
libiocml	Writer for the Chemical Markup Language format		XOM 1.2.5, CMLXOM 3.1 [91]
sdg	Structure diagram generation.	Much improved overlap resolution	
smiles	Reading and writing in the SMILES format	SMILES support performance and coverage is greatly improved	Beam 0.9.1 [66]
smarts	Substructure searching with the SMARTS format		Beam 0.9.1 [66]
hash	Molecular hash codes [92]		
formula	Chemical formula support	New	
fingerprint	Calculate fingerprints	Many new fingerprint types (see text)	Apache Commons Math 3.1.1
qsar and qsarmolecular	Molecular descriptors		XOM 1.2.5, JAMA 1.0.3 [93]
signatures	Calculation of molecular and atomic signatures		Signatures 1.1

An overview of a selection of often used CDK modules with description, dependencies on third-party libraries, and the major changes since version 1.2. Dependencies between modules are depicted in Fig. 7

#### Documentation

The quality of the JavaDocs was originally tested with DocCheck, and later replaced by a custom written tool called OpenJavaDocCheck. With the move to Maven (explained later), which does not have integration for this tool, we adopted CheckStyle (http://checkstyle.sourceforge.net/). This tool reports on missing documentation and on documentation which is not properly annotated in the Java source files. The new website lists a few resources to help starting CDK users, including a book [80] and the Chemistry Toolkit Rosetta Wiki (http://ctr.wikia.com/wiki/Chemistry_Toolkit_Rosetta_Wiki).

#### Testing

Years of development of the CDK library has resulted in a large suite of tests of various kinds. This include unit tests, which test core APIs, and functional testing, which test higher level functionality of the CDK. The latter include tests if algorithm implementations calculate the expected values, but also contain integrated tests, which involve more than one algorithm, such as SMILES parsing. The suite consists of more than 23 thousand tests.

#### Code quality

The project continues to use PMD (http://pmd.sf.net/) for code quality checking, but deviates from the default rules. For example, we are more liberal with variable name length. Moreover, a number of additional PMD tests have been developed specifically for the CDK, that, for example, test if a class uses the core interfaces instead of implementations of those interfaces. That is, that the code uses IAtom instead of Atom. However, these tests do generate a few false positives, as the tests check the class name only, and not the Java package the class is in.

#### Continuous integration

The CDK has had an automated build system for many years now. Originally, Nightly integrated various tools (building, testing, JavaDoc, etc) [2]. After the move to Maven, running various steps could be done with Maven, and Jenkins was used to execute the steps (one instance is still running at https://jenkins.bigcat.unimaas.nl/job/cdk/. The online Travis-CI service is used to build all branches, including pull requests, to ensure everything properly compiles: https://travis-ci.org/cdk/cdk.

#### Git, branching, and patches

Older versions of the CDK employed Subversion for version control. A few years back, the project switched to the Git version control system. A key advantage of this shift is the ability to have distributed repositories, easier branching and provision for patches. GitHub (https://cdk.github.io/) has replaced SourceForge as the main source code hosting service where we can use novel approaches for commenting on code (peer review), pull requests, etc. These new features simplify our code review process.

#### Support

Besides the aforementioned sources of documentation, the project has additional sources of support. First, the issue tracker welcomes questions and other types of support requests, available at https://github.com/cdk/cdk/issues. The mailing list is another place where support can be requested, while the archives document many past user questions. The list and archives can be accessed from https://sourceforge.net/p/cdk/mailman/cdk-user/.

### Binary distributions

#### Maven packages

The build system has been converted from Ant to Maven. The shift was motivated by the easier dependency handling, cleaner separation of testing code from the main library and automated packaging. The move to modules necessitated splitting the original monolithic source code tree in to per-module source folders. While this makes the on-disk layout of the source code more complex, this is usually hidden by modern IDEs.

As a result for many modules, the test code is now more closely linked to the code being tested: both reside in the same folder, though we adhere to the Maven custom to have src/main/java and a src/test/java folders. For a few modules, however, this solution introduces circular dependencies, in which case a separate Maven module is created for the tests.

The Maven packages for the CDK are available from Maven Central, which makes it easy for other projects to use. The full library can be included in other software by depending on the cdk artifact (http://search.maven.org/#search|ga|1|org.openscience) but dependencies can also be defined on individual CDK modules.

#### OSGi bundles

OSGi bundles are available for the CDK too, which are used by e.g. Bioclipse [27, 28] and KNIME [8]. However, because CDK Java packages are occasionally split between CDK modules, the CDK currently needs to be bundled as a single OSGi jar. The bundle is available from http://pele.farmbio.uu.se/bioclipse/cdk/cdk-1.5.13/. This Java package and bundle incompatibilities are currently being explored and constitutes an area where improvements can be done on modularization.

### Systematic benchmark

A systematic benchmark was performed to evaluate and quantify performance improvements from v1.4.19 to v2.0. The benchmark is divided into several cheminformatics tasks for common use cases. Each task was evaluated on input from ChEBI 149 [81] and ChEMBL 22.1 [82] as both SMILES and SDF.

The benchmark was run on Java SE 8, CentOS 7, Intel Core i7-4790 CPU @ 3.60GHz with 16 GB of RAM. The code to run the benchmark is available in Additional file 1 allowing numbers to be recorded on the reader’s system.

The results of benchmark are summarised in Tables 5 and 6. The total elapsed times are reported in Table 5, Table 6 subtracts the first tasks results (Count Heavy Atoms) to provide a comparable measure without the overhead of input read time. The throughput as molecules per minute is reported but is less accurate for very fast running tasks.

**Table 5:** Summary of systematic benchmark comparing v1.4.19 to v2.0

Benchmark	Data set		CDK v1.4.19			CDK v2.0			Improvement
			Skip	Time	Per min	Skip	Time	Per min	
countheavy	ChEBI 149	smi	2112	22.51s	108.2K	9	0.85s	2.9M	26.48
		sdf	0	7.21s	355.4K	25	3s	854.1K	2.4
	ChEMBL 22.1	smi	0	8m39.3s	193.9K	9	10.74s	9.4M	48.35
		sdf	0	3m17.29s	510.4K	0	53.27s	1.9M	3.7
rings -mark	ChEBI 149	smi	2112	22.91s	106.3K	9	1.06s	2.3M	21.61
		sdf	0	8.71s	294.2K	25	3.11s	823.9K	2.8
	ChEMBL 22.1	smi	0	8m45.78s	191.5K	9	17.09s	5.9M	30.77
		sdf	0	4m12.01s	399.6K	0	1m6.54s	1.5M	3.79
rings -sssr	ChEBI 149	smi	2112	27.4s	88.9K	9	1.43s	1.7M	19.16
		sdf	0	11.84s	216.4K	25	3.78s	677.8K	3.13
	ChEMBL 22.1	smi	0	12m4.62s	139K	9	27.16s	3.7M	26.68
		sdf	0	7m9.58s	234.4K	0	1m8.17s	1.5M	6.3
rings -all	ChEBI 149	smi	2126	45.28s	53.8K	26	1.26s	1.9M	35.94
		sdf	16	36.56s	70.1K	40	3.51s	730K	10.42
	ChEMBL 22.1	smi	88	12m40.2s	132.5K	9	24.97s	4M	30.44
		sdf	90	8m5.64s	207.4K	0	1m5.68s	1.5M	7.39
cansmi	ChEBI 149	smi	2112	36.58s	66.6K	9	1.91s	1.3M	19.15
		sdf	35	21.15s	121.1K	26	4.37s	586.3K	4.84
	ChEMBL 22.1	smi	14	14m33.86s	115.2K	9	40.84s	2.5M	21.4
		sdf	0	8m59.82s	186.6K	0	1m29.33s	1.1M	6.04
convert -ofmt smi	ChEBI 149	smi	2112	35.63s	68.4K	16	1.47s	1.7M	24.24
		sdf	35	20.91s	122.5K	25	4.55s	563.1K	4.6
	ChEMBL 22.1	smi	14	14m26.02s	116.3K	37	26.2s	3.8M	33.05
		sdf	0	8m59.38s	186.7K	1	1m12.49s	1.4M	7.44
convert -ofmt sdf	ChEBI 149	smi	2112	32.42s	75.1K	9	10.39s	234.4K	3.12
		sdf	13	17s	150.7K	25	13.96s	183.5K	1.22
	ChEMBL 22.1	smi	0	14m25.82s	116.3K	9	5m26.29s	308.6K	2.65
		sdf	1	8m51.33s	189.5K	0	6m34.5s	255.3K	1.35
convert -gen2d -ofmt sdf	ChEBI 149	smi	2112	24m28.02s	1.7K	9	35.86s	67.9K	40.94
		sdf	13	35m12.03s	1.2K	25	42.43s	60.4K	49.78
	ChEMBL 22.1	smi	0	3h27m7s	8.1K	9	17m44.64s	94.6K	11.67
		sdf	1	5h58m30s	4.7K	0	19m42.77s	85.1K	18.19
fpgen -type path	ChEBI 149	smi	2112	1m38s	24.9K	9	10.28s	236.9K	9.53
		sdf	0	2m11.03s	19.6K	25	13.03s	196.6K	10.06
	ChEMBL 22.1	smi	0	42m56.15s	39.1K	9	6m34.67s	255.2K	6.53
		sdf	0	47m5.58s	35.6K	0	7m52.32s	213.2K	5.98
fpgen -type maccs	ChEBI 149	smi	2150	1h37m35s	416	9	19.51s	124.8K	300.1
		sdf	48	1h44m17s	409	25	21.25s	120.6K	294.45
	ChEMBL 22.1	smi	214	20h24m57s	1.4K	9	13m31.21s	124.1K	90.6
		sdf	225	24h41m46s	1.1K	0	13m26.41s	124.9K	110.25
fpgen -type circ	ChEBI 149	smi	0		–	9	4.37s	557.4K	0
		sdf	0		–	25	6.81s	376.2K	0
	ChEMBL 22.1	smi	0		–	9	2m43.45s	616.1K	0
		sdf	0		–	0	3m42.01s	453.6K	0

The total elapsed *real* time was measured with the unix time utility. The throughput is reported in molecules per minute (K = thousand, M = million) as a relatable metric. This throughput was calculated by taking the total elapsed time and dividing it by the number of molecule in the dataset (42704 for ChEBI 149, and 1678393 for ChEMBL 22.1). The ChEBI SMILES input contains 2107 blank (but valid) inputs, this accounts for the majority skipped in v1.4.19. The throughput calculation was adjust to account for this

**Table 6:** Summary of systematic benchmark comparing v1.4.19 to v2.0 without read times

Benchmark	Data set		CDK v1.4.19			CDK v2.0			Improvement
			Skip	Time	Per Min	Skip	Time	Per min	
countheavy	ChEBI 149	smi	0	0s	–	0	0s	–	
		sdf	0	0s	–	0	0s	–	
	ChEMBL 22.1	smi	0	0s	–	0	0s	–	
		sdf	0	0s	–	0	0s	–	
rings -mark	ChEBI 149	smi	0	0.4s	6.1M	0	0.21s	11.6M	1.9
		sdf	0	1.5s	1.7M	0	0.11s	23.3M	13.6
	ChEMBL 22.1	smi	0	6.48s	15.5M	0	6.35s	15.9M	1
		sdf	0	54.72s	1.8M	0	13.27s	7.6M	4.1
rings -sssr	ChEBI 149	smi	0	4.89s	498.1K	0	0.58s	4.2M	8.4
		sdf	0	4.63s	553.4K	0	0.78s	3.3M	5.9
	ChEMBL 22.1	smi	0	3m25.32s	490.5K	0	16.42s	6.1M	12.5
		sdf	0	3m52.29s	433.5K	0	14.9s	6.8M	15.6
rings -all	ChEBI 149	smi	14	22.77s	107K	17	0.41s	5.9M	55.5
		sdf	16	29.35s	87.3K	15	0.51s	5M	57.5
	ChEMBL 22.1	smi	88	4m0.9s	418K	0	14.23s	7.1M	16.9
		sdf	90	4m48.35s	349.2K	0	12.41s	8.1M	23.2
cansmi	ChEBI 149	smi	0	14.07s	173.1K	0	1.06s	2.3M	13.3
		sdf	35	13.94s	183.8K	1	1.37s	1.9M	10.2
	ChEMBL 22.1	smi	14	5m54.56s	284K	0	30.1s	3.3M	11.8
		sdf	0	5m42.53s	294K	0	36.06s	2.8M	9.5
convert -ofmt smi	ChEBI 149	smi	0	13.12s	185.7K	7	0.62s	3.9M	21.2
		sdf	35	13.7s	187K	0	1.55s	1.7M	8.8
	ChEMBL 22.1	smi	14	5m46.72s	290.4K	28	15.46s	6.5M	22.4
		sdf	0	5m42.09s	294.4K	1	19.22s	5.2M	17.8
convert -ofmt sdf	ChEBI 149	smi	0	9.91s	245.8K	0	9.54s	255.3K	1
		sdf	13	9.79s	261.7K	0	10.96s	233.8K	0.9
	ChEMBL 22.1	smi	0	5m46.52s	290.6K	0	5m15.55s	319.1K	1.1
		sdf	1	5m34.04s	301.5K	0	5m41.23s	295.1K	1
convert -gen2d -ofmt sdf	ChEBI 149	smi	0	24m5.51s	1.7K	0	35.01s	69.6K	41.3
		sdf	13	35m4.82s	1.2K	0	39.43s	65K	53.4
	ChEMBL 22.1	smi	0	3h18m28s	8.5K	0	17m33.9s	95.6K	11.3
		sdf	1	5h55m13s	4.7K	0	18m49.5s	89.2K	18.9
fpgen -type path	ChEBI 149	smi	0	1m15.49s	32.3K	0	9.43s	258.3K	8
		sdf	0	2m3.82s	20.7K	0	10.03s	255.5K	12.3
	ChEMBL 22.1	smi	0	34m16.85s	49K	0	6m23.93s	262.3K	5.4
		sdf	0	43m48.29s	38.3K	0	6m59.05s	240.3K	6.3
fpgen -type maccs	ChEBI 149	smi	38	1h37m12s	418	0	18.66s	130.5K	312.6
		sdf	48	1h44m10s	410	0	18.25s	140.4K	342.5
	ChEMBL 22.1	smi	214	20h16m18s	1.4K	0	13m20.47s	125.8K	91.2
		sdf	225	24h38m29s	1.1K	0	12m33.14s	133.7K	117.8
fpgen -type circ	ChEBI 149	smi	0		–	0	3.52s	692K	
		sdf	0		–	0	3.81s	672.5K	
	ChEMBL 22.1	smi	0		–	0	2m32.71s	659.4K	
		sdf	0		–	0	2m48.74s	596.8K	

The number of records skipped and time to run the countheavy benchmark (Table 5) has been subtracted. The remaining results provides a relative comparison without accounting for the overhead of reading the input

#### Count heavy atoms

This task highlights improvements in raw read performance. Each record is read in to a resident memory connection table and the number of heavy (non-hydrogen) atoms counted by iterating over the atoms sequentially.

**Fig. 8:**
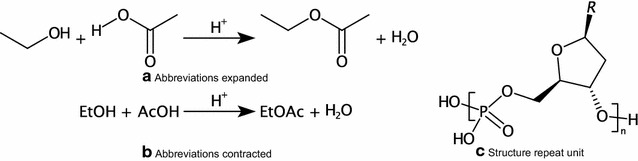
Examples of Sgroups now captured by the CDK and encoded in molfiles and CXSMILES. **a** Ethyl esterification fully expanded reaction. **b** Using Sgroup abbreviations allows display short cuts and more compact depiction. **c** An example of a structure repeat unit in DNA 5′-phosphate (CHEBI:4294)

The improvement on this task is most noticeable for SMILES input, previously it would take more than 8 min to read ChEMBL 22.1 but this is reduced to less than 11 s. On top of this improvement SMILES input is now validated and assigned a Kekulé structure. This identifies 9 invalid entries in ChEBI and another 9 in ChEMBL. Most of these rejected SMILES are due to the wrong encoding of Cis/Trans double bond stereochemistry at ring closures. The ChEBI 149 SMILES input has 2107 empty records that v1.4.19 skip, v2.0 simply reads these as empty molecules. Input from SDF also improved from ~3 to ~1 min for ChEMBL. The SDF input in v2.0 now includes perception of stereochemistry and reading CTfile Sgroups (Fig. 8). There are 9 entries from ChEBI’s SDF that are rejected because they contain CTfile query features (e.g. any bond order).

#### Rings

Ring perception is a fundamental step in many other algorithms. The rings task is divided as three subtasks: mark, sssr, and all.

*-mark* The first subtask measures the performance in marking ring membership and reporting the number of ring bonds in each record. This requires a linear algorithm based on a depth first search. The original code used a weighted spanning tree to compute the membership in linearithmic time. The run times are similar for these datasets (Table 6), larger differences are only seen for more complex cage molecules such fullerenes [38].

*-sssr* The second subtask computes the Smallest Set of Smallest Rings (SSSR) and reports the size of the SSSR (circuit rank) for each record. Although circuit rank can be computed more efficiently with a linear traversal (counting DFS back-edges) or with Euler’s polyhedron formula we are testing the time to enumerate the SSSR set. In general SSSR is considered unfavourable due to the non-uniqueness of the set and need for Gaussian matrix elimination (cubic runtime). With some bookkeeping the time spent in the matrix elimination has been reduced [38]. For ChEMBL we see the time to generate the SSSR is now ~16 s when it previously took around ~3.5 min (Table 6).

*-all* The third subtask counts the number of all rings up to or equal to size 12. This includes rings that encompass other smaller rings, for example, 1H-indole has rings of size 5, 6, and 9. In general this problem is exponential and so an adjustable threshold or timeout is used to avoid problematic molecules. CDK v1.4.19 used a timeout based threshold (default 5 s) whilst v2.0 uses a counter based on properties of algorithm [38]. In v2.0 there were 15/17^3^ records skipped from ChEBI that have complex cage-like ring systems (e.g. CHEBI:33611), no records in ChEMBL reached the threshold. By comparison in v1.4.19 there were 14/16 records skipped from ChEBI and 88/90 in ChEMBL due to reaching the time out.

The speed-up in v2.0 is slightly better than the SSSR task. ChEMBL previously spent 4–5 min and now takes only ~12–14 s (Table 6). In v2.0 finding all rings ($\le$12 bonds) runs faster than the non-unique SSSR computation.

### Canonical SMILES

This task measures the generation of a Unique SMILES string. These can be used to compare dataset intersection and exact lookup. From SMILES input v2.0 the total elapsed time is ~20 times faster for both ChEBI and ChEMBL. For ChEMBL it now takes just under 41 s to read, reorder, and write the SMILES compared to more than 14 min previously.

### Convert

This tasks tests the non-canonical conversion between SDF and SMILES input.

*-ofmt smi* SMILES is a very compact means of storing connection tables, v1.4.19 could only write canonical SMILES, v2.0 allows different SMILES flavours to be generated including a non-canonical variant. This task outputs CXSMILES that includes additional fields such as repeat groups (used by some ChEBI entries). As expected the v1.4.19 execution time is the same as for the Canonical SMILES task but v2.0 can generate the non-canonical SMILES faster taking less than 30 s for SMILES from ChEMBL.

Assigning double-bond configurations in SMILES is non-trivial and v2.0 has some safety checks, since the SMILES output is Keklué but input was aromatic, when the bond orders are assigned an extra double-bond may be accidental encoded in the SMILES output, this is sometimes acceptable but will currently report an error.

*-ofmt sdf* For writing SDF output there is minimal improvement from v1.14.19, when discounting improvements in read performance the SDF generation for ChEBI actually runs slightly slower than v1.4.19 (Table 6). This can be partially explained by the more comprehensive SDF generation that now writes Sgroups as well as computing values for atom parity and valence columns.

*-gen2d -ofmt sdf* When writing SDF the only portable way to store stereochemistry is with the inclusion of coordinates, this is specified with the -gen2d option. The overhaul in layout generation discussed early provides better layouts but also included performance tweaks, in CDK v1.4.19 generating coordinates and writing an SDF for ChEMBL took almost 3.5 h but now only takes ~18 min.

### Fingerprint generation

This task tests the generation of fingerprints for similarity and substructure screening. Three different types of fingerprints were tested, a Daylight-like Hashed Path Fingerprint, MACCS-like 166 Keys, and Pipeline Pilot-like Hashed Circular Fingerprint (ECFP4). The task generates a hexadecimal FPS file that can be used with chemfp [83].

*-type path* Path based fingerprints encode paths of length 0–7. Path based fingerprints can be used for both substructure and similarity screening. The algorithm was tweaked for v2.0 to hash the paths without pre-computing all paths upfront and without needing to generate character strings before hashing. The time to encode ChEMBL previously took 42–47 min now only takes 6–8 min.

*-type maccs* The CDK MACCS fingerprint uses 166 keys to encode features of a structure and can be used for similarity searching. This encoding uses different aspects of the library including ring perception and the new aromaticity perception but the speed is primarily dependent on SMARTS matching performance. Generating the fingerprint previously took ~1 day for ChEMBL and ~1.75 h for ChEBI. This has been reduced to less than 13.5 min for ChEMBL and ~20 s for ChEBI.

*-type circ* Circular fingerprints can only be used for similarity and could not be generated in v1.4.19. However, the fingerprints are known to perform well for retrieval performance [84]. The times are included here to show they are faster to calculate than path or MACCS-like keys and therefore recommended. CDK includes two implementations based on signatures or extended connectivity [35].

#### Benchmark summary

In all tasks, the total elapsed time is better in v2.0 compared to v1.4.19. On many tasks the improvement is more than ten times faster. Not only is the execution time improved but improvements in robustness and correctness means v2.0 is often doing much more work than the equivalent procedures in v1.4.19.

## Conclusions

Since the second CDK publication, in 2006, the library has been improved in many aspects including architecture, new functionality, improved code testing, management, peer review, and deployment. These changes have led a more functionally rich cheminformatics library, with significant performance improvements. Updates on the common SMILES and molfile formats and the improved structure diagram generation are very visible and benefit many of the tools using the CDK. Furthermore, the stability of the development model has significantly improved, providing greater stability of the library over time. With more than 90 contributors, a long list of tools based on the CDK, and hundreds of article citations, the CDK is alive and kicking.

## Availability and requirements

- *Project Name* The Chemistry Development Kit.
- *Project home page* https://cdk.github.io/.
- *Operating system(s)* Windows, GNU/Linux, OS/X.
- *Programming language* Java.
- *Other (optional) requirements* JNI-InChI, Vecmath, Beam, Guava, JGraphT, Signatures, CMLXOM, XOM, JavaCC.
- *License* LGPL v2.1 or later.
- *Any restrictions to use by non-academics* None additional.

## Additional file

**Additional file 1.** Zip file with the source code of the benchmarks.

## Footnotes

1 Molfiles can also store tetrahedral stereochemistry as a parity value, this is read if no coordinates are specified. In general there is no guarantee the parity value is read and the only portable way to store stereochemistry in a molfile is with coordinates.

2
https://github.com/cdk/cdk/commit/2fc6b61972af834c1fea7fcb64287363ecbcb188.

3 2 records from SDF use query bond features and are skipped when read.
