# Erratum to: The Chemistry Development Kit (CDK) v2.0: atom typing, depiction, molecular formulas, and substructure searching

**DOI:** 10.1186/s13321-017-0231-1

**Published:** 2017-09-20

**Authors:** Egon L. Willighagen, John W. Mayfield, Jonathan Alvarsson, Arvid Berg, Lars Carlsson, Nina Jeliazkova, Stefan Kuhn, Tomáš Pluskal, Miquel Rojas-Chertó, Ola Spjuth, Gilleain Torrance, Chris T. Evelo, Rajarshi Guha, Christoph Steinbeck

**Affiliations:** 1 Department of Bioinformatics - BiGCaT, NUTRIM, Maastricht University, 6200 MD Maastricht, The Netherlands; 2 NextMove Software Ltd, Cambridge, CB4 0EY UK; 3 Department of Pharmaceutical Biosciences, Uppsala University, 751 24 Uppsala, Sweden; 4 AstraZeneca, Innovative Medicines & Early Development, Quantitative Biology, Möndal, Sweden; 5 Ideaconsult Ltd, A. Kanchev 4, 1000 Sofia, Bulgaria; 6 Department of Informatics, University of Leicester, Leicester, UK; 7 Whitehead Institute for Biomedical Research, 455 Main Street, Cambridge, MA 02142 USA; 8 Química Clínica Aplicada, 43870 Amposta, Spain; 9 4 Hanway Place, W1T 1HD London, UK; 10 National Center for Advancing Translational Sciences, 9800 Medical Center Drive, Rockville, MD 20850 USA; 11 Institute for Inorganic and Analytical Chemistry, Friedrich-Schiller-University, Lessingstr. 8, 07743 Jena, Germany

## Erratum to: J Cheminform (2017) 9:33 DOI 10.1186/s13321-017-0220-4

After publication of this work [1], it was noticed that the graphical abstract was not included as requested.

The original article was corrected.

## Publisher’s Note

Springer Nature remains neutral with regard to jurisdictional claims in published maps and institutional affiliations.

## References

[1] Willighagen EL, Mayfield JW, Alvarsson J, Berg A, Carlsson L, Jeliazkova N (2017). The Chemistry Development Kit (CDK) v2.0: atom typing, depiction, molecular formulas, and substructure searching. J Cheminform.

